# Resveratrol as an Adjunct Therapy in Periodontal Disease: A Systematic Review and Meta-Analysis

**DOI:** 10.3390/nu18132212

**Published:** 2026-07-07

**Authors:** Suzanne Ying-Shan Su, I-Shiang Tzeng, Ting-Hsin Huang, Earl Fu

**Affiliations:** 1 Department of Dentistry, Taipei Tzu Chi Hospital, Buddhist Tzu Chi Medical Foundation, Xindian, New Taipei City 23142, Taiwan; yingshan.su@gmail.com (S.Y.-S.S.);; 2 Department of Research, Taipei Tzu Chi Hospital, Buddhist Tzu Chi Medical Foundation, New Taipei City 23142, Taiwan; 3 Department of Dentistry, School of Oral Medicine, Tri-Service General Hospital, National Defense Medical University, Taipei 114202, Taiwan

**Keywords:** resveratrol, periodontal disease, periodontitis, gingivitis, cytokine, bone loss, meta-analysis, systematic review

## Abstract

**Background/Objectives:** Resveratrol, a natural polyphenol with anti-inflammatory and antioxidant properties, has emerged as a promising adjunctive agent in periodontal therapy. This systematic review and meta-analysis evaluated evidence from in vitro, in vivo, and human clinical studies regarding the effects of resveratrol on periodontal disease, with the clinical component focusing on systemically healthy non-smoking patients. **Methods:** Electronic searches of MEDLINE/PubMed, Scopus, Embase, and Web of Science were conducted for studies published between 2000 and 2025. Eligible studies included periodontal-related in vitro cell models, in vivo experimental periodontitis models, and randomized clinical trials assessing resveratrol as an adjunctive therapy. Data extraction, risk-of-bias assessment, and meta-analyses were conducted in accordance with PRISMA guidelines. **Results:** Fifteen studies were included (five in vitro, six in vivo, and five human randomized controlled trials). Meta-analysis showed the inhibitory effect of resveratrol on the lipopolysaccharide-induced protein expression of IL-1β, IL-6, TNF-α, and IL-8 in vitro, with low to moderate heterogeneity. In animal studies, resveratrol significantly attenuated ligature-induced dental alveolar bone loss, IL-1β protein, and relative mRNA expression. However, reductions in relative mRNA expressions of TNF-α and IL-6 were inconsistent and highly heterogeneous; in contrast, nanoparticle- and liposomal-modified resveratrol consistently and significantly reduced these mRNA levels. In human trials, adjunctive resveratrol was associated with improvements in probing pocket depth and clinical attachment level compared with root planing alone in patients with periodontitis, as well as reductions in bleeding and plaque indices in patients with periodontal diseases, including gingivitis and periodontitis. **Conclusions:** Resveratrol suppresses pro-inflammatory cytokine expression in vitro and attenuates alveolar bone loss in vivo, with enhanced and more consistent molecular effects observed using modified formulations. Preliminary clinical evidence suggests that resveratrol may be associated with modest adjunctive effects on periodontal outcomes in systemically healthy non-smokers. However, given the limited number of clinical trials, small sample sizes, heterogeneity among studies, short follow-up periods, and limited certainty of the evidence, these findings should be interpreted cautiously. Further well-designed RCTs with longer follow-up periods are required to determine their clinical relevance.

## 1. Introduction

Resveratrol (3,5,4′-trihydroxy-trans-stilbene; RSV) is a natural non-flavonoid polyphenolic compound belonging to the class of stilbenes found in grapes, berries, and red wine [1,2]. It gained widespread scientific attention following the description of the “French Paradox”—the observation that French populations have relatively low incidences of cardiovascular disease in spite of diets rich in saturated fats, potentially attributed to moderate red wine consumption [3]. It has been shown to exert antioxidant, anti-inflammatory, antimicrobial, and alveolar bone-protective effects [4,5,6,7,8]. Mechanistically, resveratrol inhibits the activation of nuclear factor kappa-light-chain-enhancer of activated B cells (NF-κB) [9], modulates mitogen-activated protein kinase (MAPK) signaling [10], and activates the nuclear factor erythroid 2-related factor 2/heme oxygenase-1 (Nrf2/HO-1) pathway [11], thereby reducing oxidative stress and inflammatory cytokine production.

Gingivitis and periodontitis affect periodontal tissues and represent major inflammatory contributors to tooth loss worldwide [12,13]. These diseases arise from a dysregulated host response to the accumulation of bacterial biofilms, leading to inflammation and destruction of supporting tooth structures [14]. Gram-negative bacteria such as *Porphyromonas gingivalis* (*P.g.*) release lipopolysaccharides (LPSs), which bind to toll-like receptor 4 (TLR4) on host immune cells, triggering pro-inflammatory signaling cascades that upregulate cytokines including interleukin (IL)-1β, IL-6, IL-8, and tumor necrosis factor-alpha (TNF-α) [15]. These cytokines promote connective tissue breakdown and alveolar bone loss, perpetuating chronic inflammation [16].

Non-surgical periodontal therapy (NSPT), primarily involving mechanical debridement through scaling and root planing (SRP), remains the gold standard of periodontal disease management; however, its effectiveness can be limited under certain conditions [17]. For instance, residual bacteria embedded in tissues may not be thoroughly eliminated [17], and recurrent inflammation often impairs healing [18,19]. Adjunctive therapies have therefore been proposed to assist in biofilm control and modulate the host’s immune response [20]. Although systemic or local antibiotics are sometimes used as adjunctive therapies to SRP, concerns regarding antibiotic-resistant periodontal pathogens and potential drug interactions may limit their clinical application. In this context, natural adjunctive agents have gained increasing attention because of their biological activity, relatively low toxicity, and potential to modulate key inflammatory pathways [21].

Over recent decades, investigations into the effects of resveratrol on periodontal diseases have advanced from in vitro experiments to in vivo animal studies and, more recently, to human clinical trials. Despite encouraging findings, the available evidence remains fragmented. Accordingly, this study aims to synthesize evidence across in vitro, preclinical animal studies, and clinical investigations to provide an integrated understanding of resveratrol’s effects on periodontal disease.

## 2. Materials and Methods

### 2.1. Search Strategy

Four electronic databases—MEDLINE/PubMed, Scopus, Embase, and Web of Science—were systematically searched to identify relevant studies published from 2000 to 2025. The search strategy combined terms related to periodontal disease and resveratrol using Boolean operators and was adapted for each database using database-specific syntax. Search terms included combinations of “periodontal”, “periodontitis”, “periodontal disease”, “gingivitis”, “periodontal inflammation”, “periodontal therapy”, “resveratrol”, “trans-resveratrol”, and “3,5,4′-trihydroxy-trans-stilbene”. Controlled vocabulary terms, including MeSH terms in PubMed and Emtree terms in Embase, were used where applicable. The complete database-specific search strategies are provided in Supplementary S1. Manual searches were performed by screening the reference lists of included studies and relevant review articles and by searching Google for additional potentially eligible records. The search was limited to English-language articles, and conference abstracts were excluded. The final electronic search was performed on 31 December 2025.

All retrieved records were imported into EndNote 20 (Clarivate, Philadelphia, PA, USA), and duplicate records were removed automatically and then checked manually. A two-stage screening process (titles and abstracts first, followed by the full text) was performed by two independent reviewers (S.Y.S.S., E.F.). If there was disagreement regarding the eligibility of articles, a third reviewer (I.S.T.) was involved to resolve any conflicts through collaboration.

### 2.2. Study Selection

**Figure 1:**
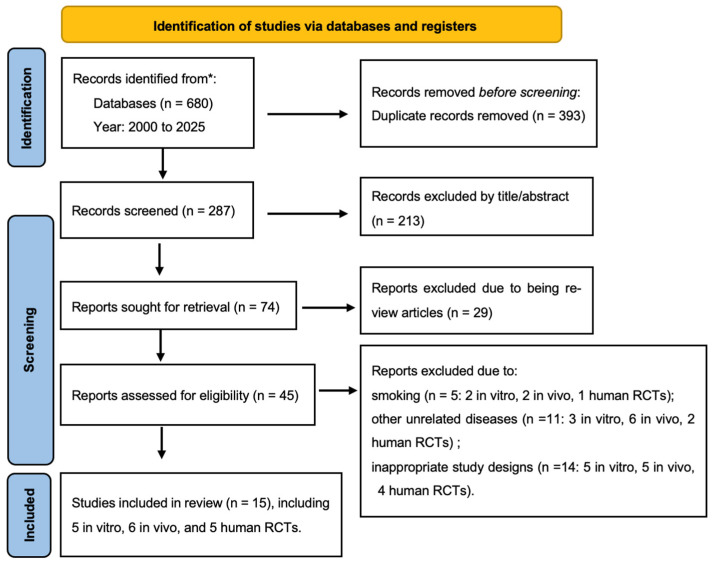
Article selection flow chart. In the present review, fifteen studies ultimately met the inclusion criteria, including 5 in vitro studies, 6 in vivo animal experiments, and 5 human RCTs, and were used for the final meta-analysis. * Records were identified from the selected electronic databases searched for the period from 2000 to 2025.

Database searches yielded 680 records covering the period from 2000 to 2025 (Figure 1). Following the removal of 393 duplicate records, 287 underwent title and abstract screening; of these, 213 were excluded for being irrelevant to the topic, leaving a total of 74 reports. Among them, 29 review articles were excluded, leaving 45 full-text articles for eligibility assessment; 30 of these were subsequently excluded because of smoking-related outcomes (*n* = 5), the presence of diseases other than periodontal diseases (*n* = 11), and methodological inconsistency with the included study designs (*n* = 14). Ultimately, a total of 15 publications were included in the qualitative synthesis. Because one publication—the study by Shi et al.—contributed both in vitro and in vivo data, these 15 publications provided 16 experimental/clinical datasets, including 5 in vitro, 6 in vivo animal, and 5 human RCT datasets. Reporting of this systematic review and meta-analysis was guided by the Preferred Reporting Items for Systematic Reviews and Meta-Analyses (PRISMA) framework, and the study protocol was registered on the Open Science Framework (OSF; DOI: https://doi.org/10.17605/OSF.IO/8HWBX) (Figure 1) before study selection and data extraction. No amendments were made after registration. The PRISMA 2020 checklist is provided in the Supplementary Material (Supplementary Table S1).

### 2.3. Focused Question

Eligible studies were categorized into in vitro, in vivo, and randomized controlled human studies (RCTs), following the PECO (Population, Exposure, Comparison, Outcome)/PICO (Population, Intervention, Comparison, Outcome) framework. For in vitro studies, PECO represented the following: P: periodontal-related cells exposed to inflammatory or bacterial stimulation; E: resveratrol treatment in any formulation or concentration; C: untreated, vehicle-treated, or stimulated cells without resveratrol; O: pro-inflammatory cytokine expression, such as IL-1β, IL-6, IL-8, and TNF-α, as well as relative mRNA or protein expression levels. For in vivo animal studies, PECO represented the following: P: animals with experimentally induced periodontal disease; E: resveratrol administration, with formulation, dose, route, frequency, and duration extracted when available; C: healthy control, untreated periodontitis model, or vehicle-treated control; O: alveolar bone loss and inflammatory markers. For human RCTs, PICO represented the following: P: those with gingivitis or periodontitis, while studies involving smokers or systemic comorbidities were excluded to reduce confounding; I: adjunctive resveratrol therapy, including oral supplements, topical gels, sprays, and mouthwashes, with formulation, dose, route, frequency, and duration extracted; C: placebo, no gel or no mouthwash, plaque removal alone, oral hygiene instruction alone, and SRP alone; O: probing pocket depth (PPD), clinical attachment level (CAL), bleeding index (BI), and O’Leary plaque index (PI).

### 2.4. Inclusion and Exclusion Criteria

Studies were included if they fulfilled one or more of the following eligibility criteria: (1) in vitro investigations utilizing periodontal- or gingival-related cell lines treated with resveratrol; (2) in vivo studies employing ligature-induced periodontitis or *P.g.*-induced murine models treated with resveratrol; (3) human RCTs evaluating gingivitis or periodontitis in which resveratrol was administered as an adjunctive therapy to NSPT. Both gingivitis and periodontitis were included because they are dental biofilm-induced periodontal diseases. Studies were excluded if they investigated other diseases or systemic confounders, enrolled smokers, lacked appropriate control groups, or employed non-comparable experimental designs.

### 2.5. Data Collection Process and Data Items

Data selection and extraction were performed independently by two reviewers (S.Y.S.S. and E.F.) using Excel (Office 365, Microsoft, Redmond, WA, USA), with a third reviewer (I.S.T.) resolving disagreements. Extracted data included the following: (1) study characteristics: author, year, country of origin, study design, experimental model, sample size, cell type, animal species, and human population characteristics; (2) intervention details: resveratrol formulation, dosage or concentration, route of administration, treatment duration, experiment/control group, and concomitant periodontal therapy; (3) primary outcomes: pro-inflammatory cytokine expression, including IL-1β, IL-6, IL-8, and TNF-α, alveolar bone loss, and relative mRNA or protein expression levels; (4) clinical outcomes in human RCTs: probing pocket depth, clinical attachment level, bleeding index, bleeding on probing, and plaque index. Plaque-related outcomes were pooled when studies reported O’Leary plaque index or equivalent full-mouth plaque percentage scores [22], as were bleeding-related outcomes when studies reported bleeding on probing, bleeding index, or full-mouth bleeding score as percentage-based bleeding outcomes [23]. Although these measures were considered clinically comparable for quantitative synthesis, differences in study population, disease status, formulation, and adjunctive periodontal therapy were recognized as potential sources of heterogeneity. Data from visual sources were estimated using WebPlotDigitizer software, version 5.2 (Automeris, https://automeris.io, accessed on 30 January 2026).) when numerical values were not directly reported. For quantitative synthesis, continuous outcomes were extracted as mean ± standard deviation. No conversion from standard error, median, interquartile range, or range to mean ± standard deviation was performed. When multiple time points were reported, the time point most comparable with other studies in the same analysis was selected. When multiple experimental arms, doses, or formulations were reported within the same study, the arm most consistent with the predefined comparison and most comparable across studies was selected for quantitative synthesis. This approach was used to reduce clinical, biological, and methodological heterogeneity. Lower values of cytokine expression, alveolar bone loss, PPD, CAL, bleeding, and plaque indices were interpreted as more favorable periodontal outcomes.

### 2.6. Risk-of-Bias Assessment

The methodological quality of each RCT was assessed using the Cochrane Risk of Bias 2 (RoB 2) tool, and five domains were evaluated: bias arising from the randomization process, bias due to deviations from intended interventions, bias due to missing outcome data, bias in outcome measurement, and bias in selection of the reported result. Based on the quality, each study was categorized into one of three groups: low risk of bias, some concerns, or high risk of bias. Animal studies were assessed using SYRCLE’s risk-of-bias tool. Because no universally accepted risk-of-bias tool exists for all cell-based in vitro studies, a structured quality and risk-of-bias framework was applied, including assessment of cell source and characterization, appropriateness of controls, exposure conditions, assay validity, replication and statistical analysis, blinding/randomization, selective reporting, and other potential sources of bias (Supplementary Table S2).

### 2.7. Certainty of Evidence

The certainty of evidence for clinical outcomes was evaluated using the GRADE approach. Evidence from randomized controlled trials was initially rated as high certainty and was downgraded according to risk of bias, inconsistency, indirectness, imprecision, and publication bias. The GRADE assessment was performed for pooled clinical outcomes, including PPD, CAL, bleeding index, and plaque index.

### 2.8. Statistical Analysis

In this meta-analysis, fifteen studies were included. Review Manager software (RevMan) version 5.4 was used for statistical analyses. For continuous outcomes, effect estimates were calculated as the mean difference (MD) or standardized mean difference (SMD), as appropriate, together with 95% confidence intervals (CIs), for parameters including cytokine levels, alveolar bone loss, probing pocket depth, clinical attachment level, plaque index, and bleeding index. MD was used when outcomes were reported using the same unit and a comparable measurement scale across studies, such as probing pocket depth and clinical attachment level measured in millimeters. SMD was used when comparable outcomes were measured using different units, scales, assays, or normalization methods, such as relative mRNA cytokine expression and protein levels. When SMD was applied, the effect size was calculated using Hedges’ g to reduce small-sample bias. Statistical significance was defined as *p* ≤ 0.05. Statistical heterogeneity was assessed using the *I*^2^ statistic and visual inspection of forest plots, with *I*^2^ values of 25%, 50%, and 75% indicating low, moderate, and high heterogeneity, respectively [24]. Model selection was not based solely on *I*^2^; clinical and methodological heterogeneity, including differences in study design, were also considered. Random-effects models were considered when clinical, biological, experimental, and methodological heterogeneity was expected, even with low *I*^2^ values. Leave-one-out sensitivity analyses were performed using R software, version 4.4.2 for outcomes with more than four included studies (Supplementary Figure S1), whereas those with fewer studies were interpreted cautiously because sensitivity analyses would be unstable.

## 3. Results

### 3.1. In Vitro Experiments (Table 1 and Figure 2)

**Figure 2:**
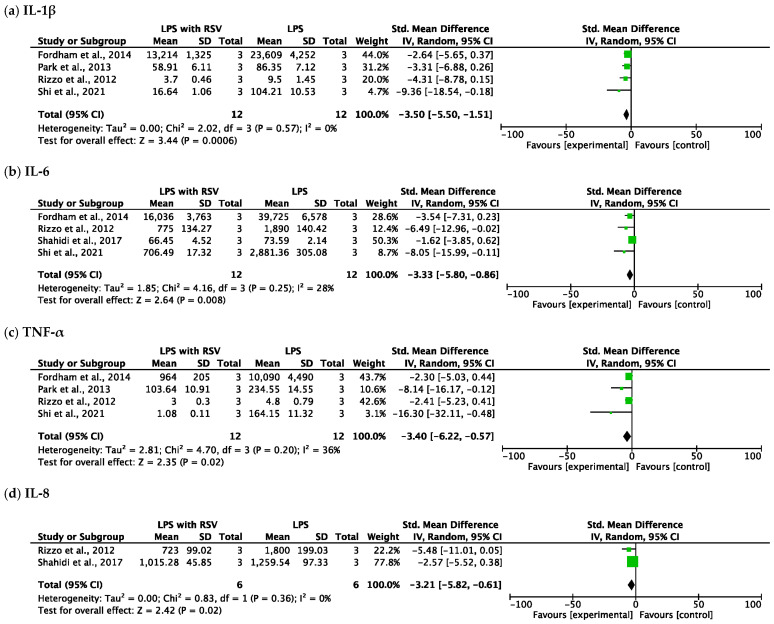
In vitro effects of resveratrol on LPS-induced pro-inflammatory cytokine protein expressions. The studies included in the forest plots were Rizzo et al., 2012 [25], Park et al., 2013 [26], Fordham et al., 2014 [27], Shahidi et al., 2017 [28], and Shi et al., 2021 [29].

Five studies assessed the in vitro effects of resveratrol on cytokine protein expressions after exposure of periodontal pathogens to LPSs (Table 1)—of which four included IL-1β, IL-6, or TNF-α, and two investigated IL-8—using a variety of cell models, including periodontal ligament cells, gingival fibroblasts, peripheral blood cells, and macrophages [25,26,27,28,29]. A structured preclinical quality/risk-of-bias framework was applied to the in vitro studies. Overall, cell source, appropriate controls, exposure conditions, and assay methods were adequately described, but blinding/randomization and selective reporting were generally unclear (Supplementary Table S2-1). Resveratrol significantly downregulated the LPS-induced IL-1β protein level (SMD = −3.50; 95% CI, −5.50 to −1.51; *p* = 0.0006; *I*^2^ = 0%) (Figure 2), and also downregulated IL-6, TNF-α, and IL-8 proteins.

**Table 1:** Summary of five in vitro resveratrol studies. Effects of resveratrol on the LPS-induced inflammatory responses were examined in cells, including human periodontal ligament cells (hPLCs), human gingival fibroblasts (hGFs), human peripheral blood mononuclear cells (hPBMCs), and murine macrophages (ΦMs). Abbreviations: IL: interleukin; COX-2: cyclooxygenase-2; PGE2: prostaglandin E2; PKC: protein kinase C; PI3K: phosphatidylinositol 3-kinase; NO: nitric oxide; ROS: reactive oxygen species; MAPK: mitogen-activated protein kinase; IFN-γ: interferon-gamma; Lipo-RSV: resveratrol-loaded liposomal system. ↑: indicates an increase; ↓: indicates a decrease; ^a^: number of experiments repeated; ^b^: the concentration or dosages selected for the meta-analysis; the underline: the cytokines included in the present meta-analysis.

Study	Country	Cell	No ^a^	Exposure	Resveratrol	Observed Effects
Rizzo et al., 2012 [25]	Italy	hPLC	3	*Pg* LPS: 1 µg/mL	25/50/100 ^b^ µM	↓ IL-1β, IL-6, IL-8, TNF-α, IL-12, NO
Park et al., 2012 [26]	South Korea	hGF	3	*Pg* LPS, 1 µg/mL; Nicotine, 0–10 mM	25 mM and/or Ad-SIRT1	↓ IL-1β, TNF-α, ROS, PGE2, COX-2, PKC, PI3K, MAPK, and NF-kB
Fordham et al., 2014 [27]	USA	hPBMC	3	*Aa* LPS: 1 µg/mL)	1 × 10^−4^ M (100 µM)	↓ IL-1β, IL-6, TNF-α, IFN-γ, IL-1α, NF-κB, and chemokines
Shahidi et al., 2017 [28]	Iran	hGF	3	*Pg* LPS: 10 µg/mL)	100 ^b^ and 200 µM	↓ IL-6, IL-8; synergistic effect with silymarin
Shi et al., 2021 [29]	China	MΦ	3	*Pg* LPS: 0.1 µg/mL	RSV ^b^, Lipo-RSV: 15 μM	↓ IL-1β, IL-6, and TNF-α, ROS, NF-κB; M1 to M2 repolarization

### 3.2. In Vivo Experiments (Table 2 and Figure 3)

**Figure 3:**
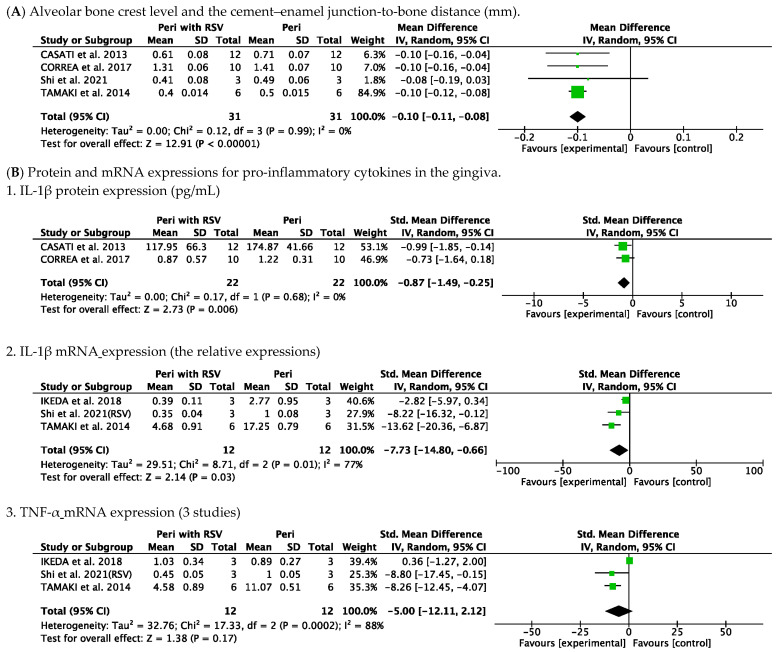

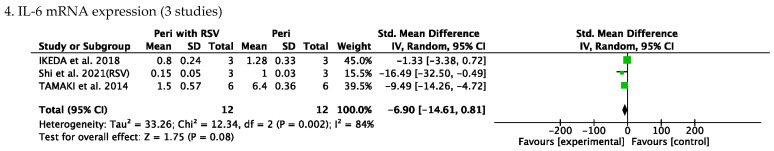
In vivo effects of resveratrol on the induced periodontitis. The studies included in the forest plots were Shi et al., 2021 [29], Tamaki et al., 2014 [30], and Ikeda et al., 2018 [31], Corrêa et al., 2017 [32], Casati et al., 2013 [33].

The in vivo effects of resveratrol reported across the six included studies are summarized in Table 2 [29,30,31,32,33,34]. Only studies that evaluated alveolar bone loss through morphometric analysis using the cement–enamel junction-to-bone crest distance in methylene blue-stained samples were included in the analysis. Among the four eligible studies [29,30,32,33], the alveolar bone crest level induced by ligation (cementoenamel junction-to-bone distance, mm) was significantly reduced after resveratrol, with no observed heterogeneity (MD = −0.10; 95% CI, −0.11 to −0.08; *p* < 0.00001; *I*^2^ = 0%) (Figure 3A).

**Table 2:** Summary of six in vivo resveratrol studies selected. Effects of resveratrol, or its modified formulations, on dental alveolar bone destruction and pro-inflammatory cytokine expressions induced by ligation (with/without *P.g.* LPS) or *P.g.*-injection. Abbreviations: Lig: ligation; RSV: resveratrol; *P.g.*: *Porphyromonas gingivalis*; ip: intra-peritoneal injection; PBS: phosphate-buffered saline; Rnp: resveratrol nanoparticle; Rnp-PPD: RSV nanoparticle loaded with 20(S)-protopanaxadiol; Sirt1: sirtuin 1; AMPK: AMP-activated protein kinase; IL: interleukin; NF-κB: nuclear factor kappa-light-chain-enhancer of activated B cells; NLRP3: NOD-like receptor family pyrin domain containing 3; Arg-1: arginase-1; TNF-α: tumor necrosis factor-alpha; TGF-β: transforming growth factor-beta; ROS: reactive oxygen species. ↑: indicates an increase; ↓: indicates a decrease; the underline: the bone loss or cytokines selected in this meta-analysis.

Author	Country	Study Design	Sample (n)	Intervention	Control	Outcomes Measured	Key Findings
Casati et al., 2013 [33]	Brazil	Wistar rats, Lig-induced	24 rats: 2 groups	RSV, Oral	Lig + Placebo	Alveolar bone loss, IL-1β, IL-4, IL-17	↓ Bone loss, IL-1β, IL-17
Tamaki et al., 2014 [30]	Japan	Wistar rats; Lig-induced	18 rats: 3 groups	Melinjo RSV, oral	1: Healthy 2: Lig	Bone loss, oxidative stress, cytokines, Nrf2/Sirt1/AMPK	↓ Bone loss, oxidative, IL-1β, TNF-α, IL-6, other cytokines;↑ antioxidants
Corrêa et al., 2017 [32]	Brazil	Wistar rats, Lig-induced	40 rats: 4 groups	RSV, Oral	Lig + Placebo	Bone loss and cytokines	↓ Bone loss and IL-1β; ↑ IL-4
Ikeda et al., 2018 [31]	Japan	C57BL/6J mice, Lig-induced	89 mice: 4 groups	Melinjo seed, ip	1: Lig 2. Lig-removed	Bone loss, oxidative stress, osteoclastogenesis	↓ Bone loss, IL-1β, TNF-α, IL-6, oxidative stress, osteoclast; ↑bone heal
Shi et al., 2021 [29]	China	Balb/c mice, Lig + *P.g.* LPS	16 mice: 4 groups	Sulcus RSV or Lipo-RSV	1: Lig + PBS 2: Minocycline	Cytokines, ROS, bone loss, NF-κB/NLRP3	↓ Bone loss, IL-1β, TNF-α, IL-6, ROS, NF-κB/NLRP3
Huangfu et al., 2023 [34]	China	SD Rat *P.g.*-injection	20 rats: 4 groups	Rnp & Rnp-PPD, injection	3: *P.g.*-induced 4: Blank	IL-1β, IL -6, IL-10, TNF-α, Arg-1, TGF-β, ROS	↓ ROS, TNF-α, IL-1β, IL-6; ↑ IL-10, Arg-1, TGF-β

Resveratrol significantly reduced IL-1β in animals with induced periodontitis at both the protein and mRNA levels (Figure 3(B1,B2)). However, the relative mRNA levels for TNF-α and IL-6 were statistically indistinguishable between the RSV and non-RSV groups (*p* = 0.17 and 0.08, respectively) (Figure 3(B3,B4)). In the study by Correa et al. [32], similar TNF-α protein levels were observed. Different from conventional resveratrol, modified formulations of liposomal RSV [29] and nanoparticle-based RSV [34] consistently demonstrated significant reductions in relative mRNA levels, regardless of whether they were for IL-1β, TNF-α, or IL-6.

SYRCLE’s risk-of-bias assessment indicated that most in vivo studies adequately described the experimental model and intervention procedures, but allocation concealment, random housing, blinding, and random outcome assessment were generally unclear (Supplementary Table S2-2).

### 3.3. Human RCT (Table 3 and Figure 4)

**Figure 4:**
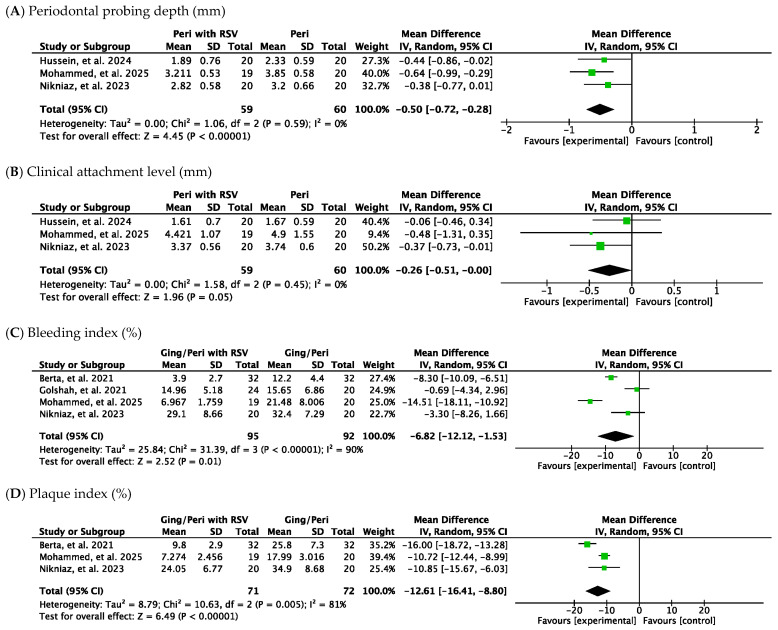
Clinical periodontal outcomes of adjunctive resveratrol in human RCTs. The studies included in the forest plots were Berta et al., 2021 [40], Golshah et al., 2021 [41], Nikniaz et al., 2023 [42], Hussein et al., 2024 [43], and Mohammed et al., 2025 [44].

Twelve RCTs were initially screened [35,36,37,38,39,40,41,42,43,44,45,46]; seven were excluded because the patients involved were diabetic [35,36], smokers [38], did not specify NSPT [37,45,46], or provided incomplete data [39]. Table 3 provides a concise review of the five included trials [40,41,42,43,44] and their risks of bias. The three included studies focused on the outcome of PPD (Figure 4A) [42,43,44] suggest adjunctive resveratrol therapy (oral administration, gel application, and mouth washing) tended to reduce the probing depth in patients with periodontitis (n = 59 and 60 for with and without resveratrol, respectively) with no heterogeneity (MD, −0.50; 95% CI, −0.72 to −0.28; *p* < 0.00001; *I*^2^ = 0%), while the reduction in CAL presented marginal significance (MD, −0.26; 95% CI, −0.51 to −0.00; *p* = 0.05, *I*^2^ = 0%) (Figure 4B). CAL was reported as the distance from the CEJ to the base of the pocket; therefore, lower post-treatment CAL values were interpreted as attachment gain or reduced attachment loss. The resveratrol interventions also tended to reduce the bleeding index (four studies included [40,41,42,44]) and plaque O’Leary index (three studies included [40,42,44]), but with high heterogeneity, in patients with gingivitis and periodontitis (Figure 4C,D).

Using the GRADE approach (Supplementary Table S3), the certainty of evidence was rated as moderate for PPD reduction and low for CAL gain, bleeding index, and plaque index. The main reasons for downgrading were the small number of studies and participants, imprecision, and substantial heterogeneity in bleeding- and plaque-related outcomes.

**Table 3:** Summary of five resveratrol RCTs. The adjunctive effects of resveratrol and its modified formulations on clinical outcomes in patients with periodontal diseases. The SRP was applied to patients with periodontitis, while the intervention of mechanical plaque control or gingival massage was applied to those with gingivitis. Abbreviations: YO: age in years old; RSV: resveratrol; RV-HPβCD: the complexation of RSV with 2-hydroxypropyl-β-CD; FMPS: full mouth plaque score; FMBS: full mouth bleeding score; GI: gingival index; HI: hyperplastic index; BOP: bleeding on probing; PPD: probing pocket depth; CAL: clinical attachment level; PI: plaque index; BI: bleeding index; IL-1β (6M) *: IL-1β measured at 6 month only. ↑: indicates an increase; ↓: indicates a decrease; the underline: the outcomes selected in this meta-analysis. Risk of bias: D1, randomisation process; D2, reviations from the intended interventions; D3, missing outcome data; D4, measurement of the outcome, and D5: selection of the reported result; 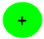 low risk; 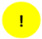 some concerns; 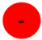 high risk.

Author	Population	Intervention	Control	Duration	Outcomes	D1	D2	D3	D4	D5	Overall
Berta et al., 2021 [40] (Italy)	N = 64, 2–5 YO children; gingivitis	RV-HPβCD spray + plaque removal	Only plaque removal	4 wks	FMPS ↓, FMBS ↓	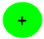	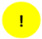	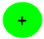	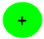	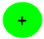	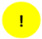
Golshah et al., 2021 [41] (Iran)	N = 69, 12–25 YO; fixed appliances, gingivitis	Gum massage with RSV emulgel	Massage with a placebo, or no gel	8 wks	GI ↓, HI ↓, BOP, PPD	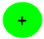	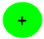	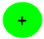	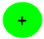	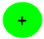	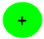
Nikniaz et al., 2023 [42] (Iran)	N = 40, 30–60 YO; periodontitis, stage II/IV, grade B	Oral RSV capsules + SRP	Placebo (starch) + SRP	4 wks	PPD, CAL, PI ↓, BI, IL-8, IL-1β	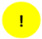	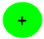	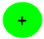	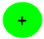	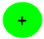	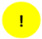
Hussein et al., 2024 [43] (Egypt)	N = 40, 26~47 YO; periodontitis, stage I/II, grade A	0.01% Topical RSV gel SRP	SRP only	3 and 6 months	PI, GI, PPD, CAL, ↓ IL-1β (6M) *	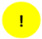	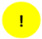	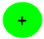	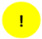	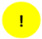	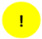
Mohammed et al., 2025 [44](Iraq)	N = 57, >18 YO; periodontitis	Nano-RSV mouthwash; CHX mouthwash; SRP	Placebo; SRP	4 wks	↓ PI, ↓ BOP, ↓PPD, CAL, ↓ IL-6	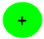	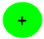	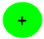	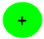	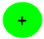	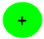

## 4. Discussion

To evaluate the potential of resveratrol as an adjunctive therapy in the management of periodontal diseases, its in vitro effects on LPS-induced cellular pro-inflammatory cytokine expression and on in vivo bony destruction and gingival expressions of pro-inflammatory cytokines, as well as the clinical outcomes after resveratrol interventions in human RCTs, were gathered, summarized, and analyzed in this meta-analysis (Figure 2, Figure 3 and Figure 4). However, given the translational nature of periodontal research, evidence from in vitro, in vivo, and clinical studies was synthesized narratively and quantitatively within their respective domains, rather than directly pooled across study types.

### 4.1. In Vitro Evidence

Resveratrol demonstrated inhibitory effects on pro-inflammatory cytokines (IL-1β, IL-6, IL-8, and TNF-α) [25,26,27,28,29] across multiple periodontal cell types, including human periodontal ligament cells, gingival fibroblasts, peripheral blood mononuclear cells, and macrophages. These findings were accompanied by modulation of key signaling pathways, including suppression of NF-κB and MAPK signaling and enhanced activation of antioxidant defenses [26,29]. The present meta-analysis further supported these findings, showing a significant reduction in cytokine protein levels, with zero to moderate heterogeneities. These results suggest the consistent anti-inflammatory potential of resveratrol at the cellular level (Figure 2). However, caution is warranted when interpreting these findings, as in vitro conditions do not fully replicate the complexity of the periodontal microenvironment.

### 4.2. In Vivo Findings

In ligature-induced periodontitis rat models, resveratrol significantly reduced alveolar bone loss, with zero heterogeneity across the four studies selected (Figure 3A) [29,30,32,33]. Six in vivo studies were initially searched; however, two were excluded from the bone-loss analysis because Ikeda et al. reported bone loss as the difference between the ligature and control sides [31], whereas Huangfu et al. did not report direct bone measurements. (Table 2) [34]. In addition, for the study by Shi et al., only measurements from the mesiolingual site were selected for analysis [29]. Mechanistically, resveratrol activates Sirt1/AMPK and Nrf2/antioxidant pathways, enhancing antioxidant enzyme activity (SOD, catalase, HO-1) and thereby neutralizing ROS while suppressing cytokine-driven osteoclastogenesis [30,32]. Given that IL-1β, IL-6, and TNF-α upregulate RANKL expression in osteoblasts, driving osteoclast activation and bone resorption, resveratrol’s dual action on inflammation and oxidative stress interrupts this destructive cycle [29,33,47,48]. In addition to the reduction in alveolar bone loss observed in our analysis, other studies have shown resveratrol acts as a modulator of osteogenesis by counteracting TCDD (2,3,7,8-tetrachlorodibenzo-p-dioxin)-induced impairment of osteoblast differentiation [8,49], promoting mesenchymal stem cell osteogenesis via the SIRT1 and FOXO3A [50] axis, and enhancing maturation of pre-osteoblasts [51,52,53]. Future periodontal studies should therefore evaluate osteogenic markers and regenerative endpoints to clarify whether resveratrol contributes to bone formation beyond its anti-inflammatory and bone loss-reducing effects.

However, the transcriptional and translational responses to resveratrol were varied in the selected animal studies regarding the expression of individual pro-inflammatory cytokines: resveratrol significantly reduced IL-1β mRNA and protein levels (Figure 3(B1,B2)), but did not influence the levels of TNF-α or IL-6 mRNA (Figure 3(B3,B4)), or TNF-α protein [32]. This inconsistency may reflect differences in post-transcriptional regulation, experimental timing, or variability in resveratrol bioavailability across studies. Moreover, unlike conventional resveratrol, studies using modified resveratrol formulations consistently showed significant reductions in the relative mRNA expression of IL-1β, TNF-α, and IL-6 [29,34]. Due to the limited number of studies, formal subgroup analyses were not feasible; nevertheless, further detailed investigation is indicated. The favorable effects of modified resveratrol formulations may be partly related to enhanced local bioavailability, as these formulations can help overcome the low water solubility and extensive systemic metabolism of conventional resveratrol [54,55,56,57]. Beyond improving its low aqueous solubility and systemic bioavailability, these delivery systems may protect resveratrol from rapid degradation, prolong local retention, enhance penetration into periodontal tissues, and improve cellular uptake [54,55,56,57]. Controlled or sustained release may also help maintain more stable local concentrations at inflamed periodontal sites, which could partly explain the more consistent anti-inflammatory effects observed with modified formulations. However, direct comparative evidence between conventional and modified resveratrol formulations remains limited; therefore, these findings should be interpreted cautiously and require confirmation in future studies.

It should be emphasized that animal models do not fully replicate clinical periodontal therapy conditions, particularly the absence of mechanical debridement (SRP), which may limit direct translation to human outcomes. In the included animal studies, resveratrol was generally tested in experimentally induced periodontitis without mechanical plaque removal or scaling and root planing. In contrast, the human RCTs evaluated resveratrol as an adjunct to NSPT/SRP or plaque control. Therefore, the effects observed in untreated experimental periodontal lesions are not directly comparable to those of adjunctive resveratrol use following periodontal debridement in humans. These animal findings should be interpreted primarily as evidence of biological plausibility, rather than direct support for clinical efficacy, and future animal studies incorporating mechanical periodontal treatment models may help clarify whether resveratrol provides additional benefits beyond plaque removal or SRP.

### 4.3. Human Clinical Implications

The present meta-analysis of the human RCTs suggests that resveratrol, when used as an adjunctive therapy to NSPT, may be associated with modest favorable changes in selected periodontal outcomes. Adjunctive resveratrol was associated with a statistically significant but modest reduction in PPD, while the effect on CAL was only marginally favorable, alongside reductions in bleeding and O’Leary plaque index scores (Figure 4). Although no statistical heterogeneity was detected for PPD and CAL (*I*^2^ = 0%), this finding should be interpreted cautiously because of the limited number of included trials and small sample sizes. In contrast, substantial heterogeneity was observed for the bleeding and plaque indices, suggesting that differences in study design, patient populations, follow-up duration, and resveratrol formulations may have influenced the pooled estimates. Although the exact reasons for the high heterogeneity were not explored in this analysis, differences in disease severity may partly explain it: the clinical trials assessing PPD and CAL were limited to patients with periodontitis [42,43,44], while trials evaluating bleeding and plaque indices were extended to children/young orthodontic patients with gingivitis [40,41]. Moreover, variations in delivery routes—oral administration, gel, spray, and mouthwash—between studies [40,41,42,43,44] may have further contributed to outcome variability.

Among the five randomized clinical trials, three employed modified formulations to improve resveratrol bioavailability and local delivery [40,41,44]: Berta et al. reported that a nanoencapsulated resveratrol formulation complexed with 2-hydroxypropyl- β-cyclodextrin significantly reduced plaque and bleeding indices [40]; Golshah et al. showed that a resveratrol-containing emulgel, a lipophilic compound, improved gingival and hyperplastic indices by enhancing solubility and mucosal penetration [41]; Mohammed et al. further reported that a nanotechnology-enhanced resveratrol mouthwash improve the plaque index, bleeding on probing, probing pocket depth, and salivary IL-6 levels [44].

In contrast, the other two studies did not use modified formulations. Nikniaz et al. observed only a significant reduction in plaque index, which may partly reflect poor oral bioavailability of resveratrol [42], as it undergoes extensive first-pass metabolism, primarily through glucuronidation and sulfation, resulting in low systemic bioavailability of the active compound [55]. Moreover, Hussein et al. reported a significant reduction in IL-1β at 6 months without improvements in other periodontal parameters, which may be related to the use of a simple hydrophilic gel despite resveratrol’s poor aqueous solubility [43].

Nevertheless, after pooling these studies, our analysis indicates a consistent trend toward potential clinical improvement with adjunctive resveratrol, including PPD reductions and modest CAL gain in patients with periodontitis, as well as reductions in bleeding and plaque indices in patients with periodontitis and gingivitis (Figure 4). Resveratrol exhibits antimicrobial effects, including inhibition of biofilm formation, membrane disruption, and downregulation of virulence genes, which may contribute to decreased plaque burden [58,59]. Its modulation of intracellular signaling pathways, such as TLR4 and ERK/Wnt, may further attenuate inflammatory responses and tissue destruction [60,61]. However, the human evidence remains limited to a few small-scale clinical trials with modest sample sizes and short study durations, restricting the generalizability of the findings.

### 4.4. Limitations

Several limitations should be acknowledged. Fewer than five studies were available per analysis across in vitro, in vivo, and human RCT studies, which limits the power to detect heterogeneity and precludes a reliable assessment of publication bias. Although Hedges’ g was used to reduce small-sample bias, the pooled effect size should be interpreted with caution.

Moreover, in vitro and in vivo studies cannot fully reflect the true clinical condition, as SRP cannot be performed in animal models; therefore, the alveolar bone loss pattern may not directly correspond to clinical outcomes such as PPD or CAL observed in human RCTs. High heterogeneity was noted in preclinical animal models, which may be attributed to variations in animal species, resveratrol sources, delivery routes, and dosage regimens. The available human evidence is limited to a few small-scale clinical trials with modest sample sizes and short study durations, which constrains the generalizability of the findings. Moreover, the English-language restriction, potential risk of unit-of-analysis errors in preclinical experiments, limited generalizability due to the exclusion of smokers and diabetic patients, and indirectness of translating in vitro and animal findings to clinical practice may also restrict the interpretation of the results.

## 5. Conclusions

Within the limitations of the available evidence, this systematic review and meta-analysis suggests that resveratrol, or its modified formulations, consistently reduced pro-inflammatory cytokine expression in vitro and attenuated ligature-induced alveolar bone loss in vivo. However, cytokine expression with conventional resveratrol delivery in animals was inconsistent; in contrast, the modified formulations consistently reduced expression. Preliminary clinical evidence in systemically healthy non-smokers suggests that adjunctive resveratrol is associated with statistically significant but modest improvements in selected periodontal parameters, particularly PPD, while CAL gain is small. Given the limited number of RCTs, small sample sizes, heterogeneity in study design and delivery formulations, short follow-up periods, and limited certainty of the evidence, the present findings should be interpreted cautiously. Current evidence supports resveratrol as a promising investigational adjunctive therapy rather than an established periodontal therapy, and larger, well-standardized randomized controlled trials with longer follow-up are needed to confirm whether these effects translate into clinically meaningful periodontal benefits.

## Data Availability

All data generated or analyzed in this review are included in this published article.

## Supplementary Materials

The following supporting information can be downloaded at https://www.mdpi.com/article/10.3390/nu18132212/s1. Supplementary Table S1. PRISMA 2020 checklist; Supplementary Table S2. In vitro and in vivo risk of bias assessment; Supplementary Table S3. Certainty of evidence for human RCT outcomes using GRADE; Supplementary S1. Detailed search terms; Supplementary Figure S1. Results of Leave-one-out sensitivity analyses.

## Abbreviations

IL	Interleukin
TNF-α	Tumor necrosis factor-alpha
NF-κB	Nuclear factor kappa-light-chain-enhancer of activated B cells
Nrf2	Nuclear factor erythroid 2-related factor 2
HO-1	Heme oxygenase-1
MAPK	Mitogen-activated protein kinase
*P.g.*	*Porphyromonas gingivalis*
LPS	Lipopolysaccharide
NSPT	Non-surgical periodontal therapy
SRP	Scaling and root planing
RCTs	Randomized controlled human studies
PICO	Population, intervention, comparison, outcome
RoB 2	Cochrane risk of bias 2
RevMan	Review manager
MD	Mean difference
SMD	Standardized mean difference
CIs	Confidence intervals
PPD	Probing pocket depth
CAL	Clinical attachment level
hPLCs	Human periodontal ligament cells
RSV	Resveratrol
hGFs	Human gingival fibroblasts
hPBMCs	Human peripheral blood mononuclear cells
ΦMs	Murine macrophages
COX-2	Cyclooxygenase-2
PGE2	Prostaglandin E2
PKC	Protein kinase C
PI3K	Phosphatidylinositol 3-kinase
Lipo-RSV	Resveratrol-loaded liposomal system
Lig	Ligation
Rnp	Resveratrol nanoparticle
Rnp-PPD RSV	Nanoparticle loaded with 20(S)-protopanaxadiol
PBS	Phosphate-buffered saline
Sirt1	Sirtuin 1
AMPK	AMP-activated protein kinase
NLRP3	NOD-like receptor family pyrin domain containing 3
Arg-1	Arginase-1
TGF-β	Transforming growth factor-beta
ROS	Reactive oxygen species
