# Supplementary material for: Resveratrol as an Adjunct Therapy in Periodontal Disease: A Systematic Review and Meta-Analysis
Source: Nutrients. 2026 Jul 7;18(13):2212. doi: 10.3390/nu18132212 (PMC13363819; doi:10.3390/nu18132212)

SUPPLEMENTARY MATERIALS

Title

Resveratrol as an Adjunct Therapy in Periodontal Disease: A Systematic Review and Meta-Analysis

Authors

Suzanne Ying-Shan Su [1], I-Shiang Tzeng [2], Ting-Hsin Huang [1], Earl Fu [1 and 3]

Affiliations

1. Department of Dentistry, Taipei Tzu Chi Hospital, Buddhist Tzu Chi Medical Foundation, Xindian, New Taipei City, Taiwan; yingshan.su@gmail.com (S. Y. S. S.)
2. Department of Research, Taipei Tzu Chi Hospital, Buddhist Tzu Chi Medical Foundation, New Taipei City, Taiwan
3. Department of Dentistry, School of Oral Medicine, Tri-Service General Hospital, National Defense Medical University, Taipei, Taiwan

TABLE OF CONTENTS

Supplementary Table S1. PRISMA 2020 checklist

Supplementary S1. Detailed search terms

Supplementary Table S2. In vitro and in vivo risk of bias assessment

1. In vitro quality / risk-of-bias assessment

2. SYRCLE’s risk-of-bias tool for in vivo studies

Supplementary Table S3. Certainty of evidence for human RCT outcomes using GRADE

Supplementary Figure S1. Results of Leave-one-out sensitivity analyses

Table S1. PRISMA 2020 checklist

Section and Topic	Item #	Checklist item	Location where item is reported
**TITLE**			
Title	1	Identify the report as a systematic review.	Page1
**ABSTRACT**			
Abstract	2	See the PRISMA 2020 for Abstracts checklist.	Page 1,2
**INTRODUCTION**			
Rationale	3	Describe the rationale for the review in the context of existing knowledge.	Page 2
Objectives	4	Provide an explicit statement of the objective(s) or question(s) the review addresses.	Page 2
**METHODS**			
Eligibility criteria	5	Specify the inclusion and exclusion criteria for the review and how studies were grouped for the syntheses.	Page 4-5: Section 2.4
Information sources	6	Specify all databases, registers, websites, organisations, reference lists and other sources searched or consulted to identify studies. Specify the date when each source was last searched or consulted.	Page 3: Section 2.1
Search strategy	7	Present the full search strategies for all databases, registers and websites, including any filters and limits used.	Page 3-6 Supplementary

Section and Topic	Item #	Checklist item	Location where item is reported
Selection process	8	Specify the methods used to decide whether a study met the inclusion criteria of the review, including how many reviewers screened each record and each report retrieved, whether they worked independently, and if applicable, details of automation tools used in the process.	Page 3-6
Data collection process	9	Specify the methods used to collect data from reports, including how many reviewers collected data from each report, whether they worked independently, any processes for obtaining or confirming data from study investigators, and if applicable, details of automation tools used in the process.	Page 5<br>Section 2.5
Data items	10a	List and define all outcomes for which data were sought. Specify whether all results that were compatible with each outcome domain in each study were sought (e.g. for all measures, time points, analyses), and if not, the methods used to decide which results to collect.	Page 5<br>Section 2.5
	10b	List and define all other variables for which data were sought (e.g. participant and intervention characteristics, funding sources). Describe any assumptions made about any missing or unclear information.	Page 5<br>Section 2.5
Study risk of bias assessment	11	Specify the methods used to assess risk of bias in the included studies, including details of the tool(s) used, how many reviewers assessed each study and whether they worked independently, and if applicable, details of automation tools used in the process.	Page 5<br>Section 2.6<br>Supplementary

Section and Topic	Item #	Checklist item	Location where item is reported
Effect measures	12	Specify for each outcome the effect measure(s) (e.g. risk ratio, mean difference) used in the synthesis or presentation of results.	Page 6 Section 2.8
Synthesis methods	13a	Describe the processes used to decide which studies were eligible for each synthesis (e.g. tabulating the study intervention characteristics and comparing against the planned groups for each synthesis (item #5)).	Page 6
	13b	Describe any methods required to prepare the data for presentation or synthesis, such as handling of missing summary statistics, or data conversions.	Page 6
	13c	Describe any methods used to tabulate or visually display results of individual studies and syntheses.	Page 6
	13d	Describe any methods used to synthesize results and provide a rationale for the choice(s). If meta-analysis was performed, describe the model(s), method(s) to identify the presence and extent of statistical heterogeneity, and software package(s) used.	Page 6
	13e	Describe any methods used to explore possible causes of heterogeneity among study results (e.g. subgroup analysis, meta-regression).	Page 6
	13f	Describe any sensitivity analyses conducted to assess robustness of the synthesized results.	Page 6

Section and Topic	Item #	Checklist item	Location where item is reported
Reporting bias assessment	14	Describe any methods used to assess risk of bias due to missing results in a synthesis (arising from reporting biases).	Page 5-6
Certainty assessment	15	Describe any methods used to assess certainty (or confidence) in the body of evidence for an outcome.	Page 5-6 Section 2.7
**RESULTS**			
Study selection	16a	Describe the results of the search and selection process, from the number of records identified in the search to the number of studies included in the review, ideally using a flow diagram.	Page 4<br>Figure 1
	16b	Cite studies that might appear to meet the inclusion criteria, but which were excluded, and explain why they were excluded.	Page 6-12
Study characteristics	17	Cite each included study and present its characteristics.	Page 6-12
Risk of bias in studies	18	Present assessments of risk of bias for each included study.	Page 6-12<br>Supplementary
Results of individual studies	19	For all outcomes, present, for each study: (a) summary statistics for each group (where appropriate) and (b) an effect estimate and its precision (e.g. confidence/credible interval), ideally using structured tables or plots.	Page 6-12
Results of syntheses	20a	For each synthesis, briefly summarise the characteristics and risk of bias among contributing studies.	Page 6-12

Section and Topic	Item #	Checklist item	Location where item is reported
	20b	Present results of all statistical syntheses conducted. If meta-analysis was done, present for each the summary estimate and its precision (e.g. confidence/credible interval) and measures of statistical heterogeneity. If comparing groups, describe the direction of the effect.	Page 6-12
	20c	Present results of all investigations of possible causes of heterogeneity among study results.	Page 6-12
	20d	Present results of all sensitivity analyses conducted to assess the robustness of the synthesized results.	Supplementary
Reporting biases	21	Present assessments of risk of bias due to missing results (arising from reporting biases) for each synthesis assessed.	Page 6-12<br>Supplementary
Certainty of evidence	22	Present assessments of certainty (or confidence) in the body of evidence for each outcome assessed.	Page 10, Supplementary
**DISCUSSION**			
Discussion	23a	Provide a general interpretation of the results in the context of other evidence.	Page 12-15
	23b	Discuss any limitations of the evidence included in the review.	Page 12-15
	23c	Discuss any limitations of the review processes used.	Page 12-15
	23d	Discuss implications of the results for practice, policy, and future research.	Page 12-15
**OTHER INFORMATION**			

Section and Topic	Item #	Checklist item	Location where item is reported
Registration and protocol	24a	Provide registration information for the review, including register name and registration number, or state that the review was not registered.	Page 3<br>Section 2.2
	24b	Indicate where the review protocol can be accessed, or state that a protocol was not prepared.	Page 3<br>Section 2.2
	24c	Describe and explain any amendments to information provided at registration or in the protocol.	Page 3<br>Section 2.2
Support	25	Describe sources of financial or non-financial support for the review, and the role of the funders or sponsors in the review.	Page 16
Competing interests	26	Declare any competing interests of review authors.	Page 16
Availability of data, code and other materials	27	Report which of the following are publicly available and where they can be found: template data collection forms; data extracted from included studies; data used for all analyses; analytic code; any other materials used in the review.	Page 17-20

S1. Detailed search terms

PubMed:

("Periodontal Diseases"[Mesh] OR "Periodontitis"[Mesh] OR "Gingivitis"[Mesh] OR periodontal[tiab] OR periodontitis[tiab] OR "periodontal disease"[tiab] OR "periodontal diseases"[tiab] OR gingivitis[tiab] OR "periodontal inflammation"[tiab]) AND ("Resveratrol"[Mesh] OR resveratrol[tiab] OR "trans-resveratrol"[tiab] OR "3,5,4'-trihydroxy-trans-stilbene"[tiab] OR "3,5,4'-trihydroxystilbene"[tiab] OR "3,4',5-trihydroxystilbene"[tiab])

Scopus:

TITLE-ABS-KEY (periodontal OR periodontitis OR "periodontal disease" OR "periodontal diseases" OR gingivitis OR "periodontal inflammation" OR "periodontal therapy") AND TITLE-ABS-KEY (resveratrol OR "trans-resveratrol" OR "3,5,4'-trihydroxy-trans-stilbene"OR "3,5,4'-trihydroxystilbene" OR "3,4',5-trihydroxystilbene")

Embase:

('periodontal disease'/exp OR 'periodontitis'/exp OR 'gingivitis'/exp OR periodontal:ti,ab,kw OR periodontitis:ti,ab,kw OR 'periodontal disease':ti,ab,kw OR 'periodontal diseases':ti,ab,kw OR gingivitis:ti,ab,kw OR 'periodontal inflammation':ti,ab,kw OR 'periodontal therapy':ti,ab,kw) AND ('resveratrol'/exp OR resveratrol:ti,ab,kw OR 'trans resveratrol':ti,ab,kw OR 'trans-resveratrol':ti,ab,kw OR '3,5,4 trihydroxy trans stilbene':ti,ab,kw OR '3,5,4-trihydroxystilbene':ti,ab,kw OR '3,4,5 trihydroxystilbene':ti,ab,kw)

Web of Science:

TS=(periodontal OR periodontitis OR "periodontal disease" OR "periodontal diseases"OR gingivitis OR "periodontal inflammation" OR "periodontal therapy")AND TS=(resveratrol OR "trans-resveratrol" OR "3,5,4'-trihydroxy-trans-stilbene"OR "3,5,4'-trihydroxystilbene" OR "3,4',5-trihydroxystilbene")

Table S2 Risk of bias for in vitro and in vivo studies.

1. In vitro quality / risk-of-bias assessment

Study	Cell source / characterization	Appropriate controls	Exposure details: dose, time, formulation	Assay validity	Replication / statistics	Blinding / randomization	Selective reporting	Other bias / limitations	Overall concern
Rizzo et al., 2012	Low	Low	Low	Low	Unclear	Unclear	Unclear	Moderate concern: cells were isolated from one donor, which may limit biological variability	Some concern
Park et al., 2012	Low	Low	Low	Low	Low	Unclear	Unclear	Some concern:blinding/randomization not reported	Some concern
Fordham et al., 2014	Low	Low	Low	Low	Moderate concern	Unclear	Unclear	Moderate concern: low number of biological replicates noted by authors	Some concern
Shahidi et al., 2017	Low	Unclear	Low	Low	Low	Unclear	Unclear	Some concern: vehicle-control details were not fully clear	Some concern
Shi et al., 2021	Low	Low	Low	Low	Unclear	Unclear	Unclear	Some concern: detailed nanocarrier characterization was reported, but blinding/randomization for in vitro assays was not described	Some concern

2. SYRCLE's risk-of-bias tool for in vivo studies

Study	Sequence generation	Baseline characteristics	Allocation concealment	Random housing	Blinding of caregivers /investigators	Random outcome assessment	Blinded outcome assessment	Incomplete outcome data	Selective reporting	Other bias
Casati et al., 2013	Low	Low	Unclear	Unclear	Unclear	Unclear	Low	Low	Unclear	Low
Tamaki et al., 2014	Low	Low	Unclear	Unclear	Unclear	Unclear	Unclear	Low	Unclear	Low
Correa et al., 2017	Low	Low	Unclear	Unclear	Unclear	Unclear	Unclear	Low	Unclear	Unclear
Ikeda et al., 2018	Low	Low	Unclear	Unclear	Unclear	Unclear	Unclear	Low	Unclear	Low
Shi et al., 2021	Unclear	Unclear	Unclear	Unclear	Unclear	Unclear	Unclear	Unclear	Unclear	Unclear
Huangfu et al., 2023	Low	Unclear	Unclear	Unclear	Unclear	Unclear	Unclear	Unclear	Unclear	Unclear

Table S3. Certainty of evidence for human RCT outcomes using GRADE

Outcome	Studies / participants	Effect estimate	Main reasons for downgrading	Certainty of evidence	Summary interpretation
PPD reduction	3 RCTs / 119 participants	MD −0.50 mm; 95% CI −0.72 to −0.28; $I^2 = 0\%$	Imprecision due to small number of studies and participants	Moderate	Adjunctive resveratrol probably results in a modest reduction in PPD in patients with periodontitis.
CAL gain / reduction in CAL values	3 RCTs / 119 participants	MD −0.26 mm; 95% CI −0.51 to −0.00; $I^2 = 0\%$	Imprecision; small effect size; confidence interval close to the null effect	Low	Adjunctive resveratrol may result in small CAL gain, but the clinical relevance is uncertain.
Bleeding index	4 RCTs / 187 participants	MD −6.82; 95% CI −12.12 to −1.53; $I^2 = 90\%$	Serious inconsistency due to high heterogeneity and mixed gingivitis/periodontitis populations	Low	Adjunctive resveratrol may reduce bleeding indices, but the estimate should be interpreted cautiously.
Plaque index	3 RCTs / 143 participants	MD −12.61%; 95% CI −16.41 to −8.80; $I^2 = 81\%$	Serious inconsistency due to high heterogeneity, mixed populations, and different delivery formulations	Low	Adjunctive resveratrol may reduce plaque index scores, but the estimate should be interpreted cautiously.

RCT evidence was initially rated as high certainty and downgraded according to the GRADE domains: risk of bias, inconsistency, indirectness, imprecision, and publication bias. PPD and CAL analyses were limited to periodontitis studies, whereas bleeding and plaque outcomes included gingivitis and periodontitis studies. Publication bias could not be reliably assessed because fewer than 10 studies were included per outcome.

Figure S1. Results of Leave-one-out sensitivity analyses

1. IL-1β

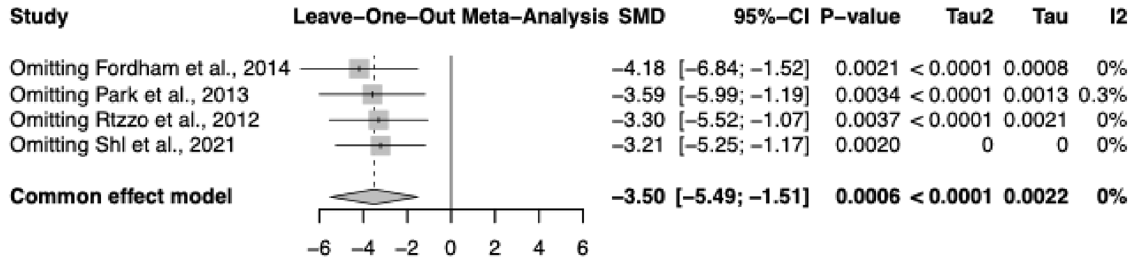

2. IL-6

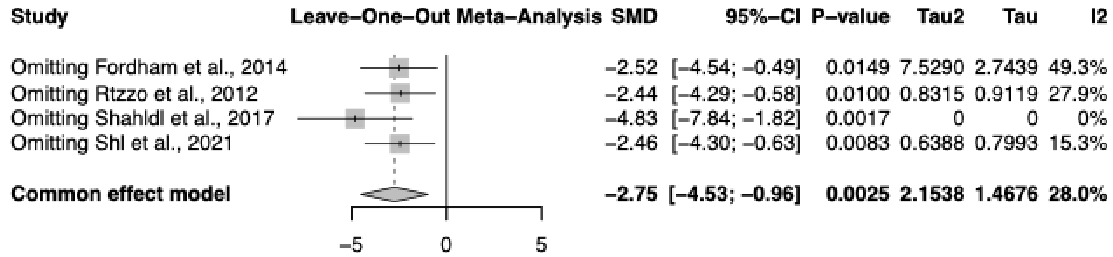

3. TNF-α

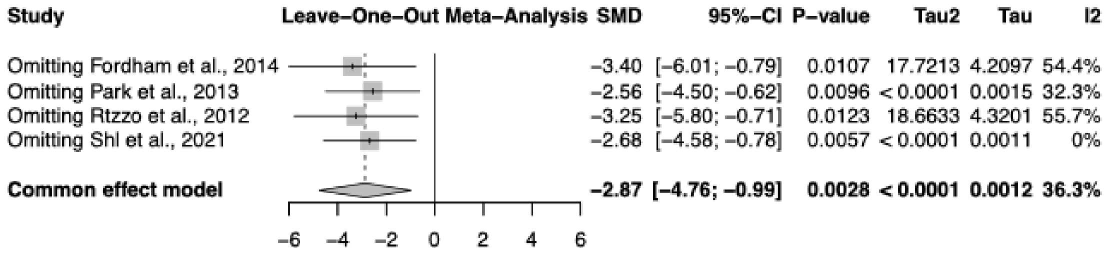

4. Alveolar bone loss

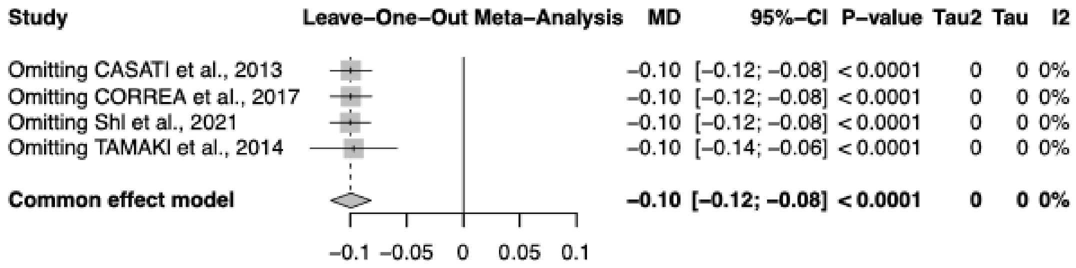

5. Bleeding index

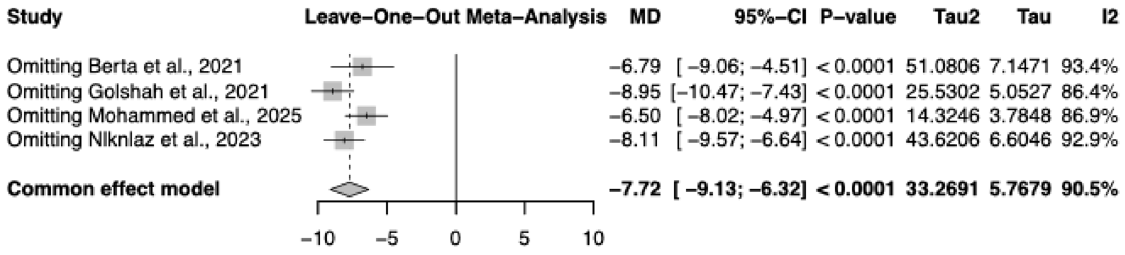

Supplement: Supplementary file 1 [file nutrients-18-02212-s001.zip › nutrients-4327130-supplementary.pdf]
